# Epidemiological Characteristics of Novel Influenza A (H1N1) in Antiviral Drug Users in Korea

**DOI:** 10.1371/journal.pone.0047634

**Published:** 2012-10-17

**Authors:** Kyunghi Choi, Sung-il Cho, Masahiro Hashizume, Ho Kim

**Affiliations:** 1 Department of Insurance Benefits, National Health Insurance Cooperation, Seoul, Republic of Korea; 2 Graduate School of Public Health and Institute of Health and Environment, Seoul National University Seoul National University, Seoul, Republic of Korea; 3 Institute of Tropical Medicine and the Global Center of Excellence Program, Nagasaki University, Nagasaki, Japan; University of Calgary & ProvLab Alberta, Canada

## Abstract

Soon after the first novel influenza A (H1N1) death was documented in Korea on August 15, 2009, prompt treatment with antiviral drugs was recommended when an infection was suspected. Free antiviral drugs were distributed to patients who met the case definition in the treatment guidelines, and patients prescribed the antiviral drugs were included in the Antiviral Drug Surveillance System (ADSS). A total of 2,825,821 patients were reported to the ADSS from September 1 to December 31, 2009. Odds ratios were calculated to compare the risks of severe diseases, as indicated by general hospital admissions or intensive care unit (ICU) admissions according to demographic characteristics, underlying medical conditions, and behavioral factors. Approximately 6% of the total population received antiviral drugs during the study period. Of these, 2,709,611 (95.9%) were outpatients, 114,840 (4.06%) were hospitalized, and 1,370 (0.05%) were admitted to the ICU. Children aged 0–9 yr accounted for 33.94% of all reported cases, whereas only 3.89% of the patients were ≥ 60 yr. The estimated incidence of novel influenza A (H1N1) during the pandemic was 5.68/100 of all reported cases. Mortality due to influenza A (H1N1) during the pandemic was 0.33/100,000, with the highest mortality of 1.31/100,000 for patients aged ≥ 60 years. Severe pandemic H1N1 influenza was associated with the presence of one or more underlying medical conditions in elderly aged ≥ 60 years and with lower economic status. Moreover, influenza A (H1N1) appeared to be age-specific in terms of mortality. Although the incidence and admission rates of influenza A (H1N1) were higher in younger age groups, fatal cases were much more likely to occur in the elderly (≥60 years). In contrast to earlier influenza A (H1N1) reports, the risks of a severe outcome were elevated among those who were underweight (body mass index < 18.5 kg/m^2^).

## Introduction

The first confirmed case of novel influenza A (H1N1) in Korea was registered on May 1, 2009 [1], and 225 deaths had been reported by January 1, 2010 [2]. Soon after the first death was documented on August 15, 2009, the Korean Health Authority revised the national guidelines so that confirmed tests would no longer be required, and general and prompt treatment with antiviral drugs was recommended as soon as the infection was suspected. The reformed guidelines were published on September 1, 2009 [3].

The Ministry of Health and Welfare in Korea stopped reporting the number of confirmed cases on September 22, 2009, because these data clearly did not represent a true picture of the pandemic [4]. Even the World Health Organization (WHO) stopped reporting confirmed cases after July 6, 2009 [5]. Every country has a different healthcare system and a different overall socioeconomic status, which made accessibility and lab capability of the testing data unequal. Accuracy of test results depends on the timing of the samples taken, and some tests are not entirely reliable [6]. Moreover, testing is not necessary in most cases.

Korea operated the Antiviral Drug Surveillance System (ADSS) nationally to monitor the use of antiviral drugs such as oseltamivir or zanamivir. All hospitals and pharmacies administering or dispensing these drugs were instructed to enter information pertaining to the prescriptions into the ADSS, a web-based system. In this report, we describe the epidemiological characteristics of all nationally representative patients in the ADSS from September 1 to December 31, 2009. This is the first study in Korea using nationwide surveillance. We also investigated social and behavioral factors correlated with illness severity from novel influenza A (H1N1).

## Methods

### Data Collection

The ADSS began to be used on September 21, 2009. The Korean Health Authority distributed antiviral drugs beginning on August 21, 2009, and the use of antiviral drugs prior to the ADSS was also entered by each medical institute. Patients who met the case definition in the treatment guidelines were supplied free antiviral drugs from national storage repositories through 455 hospitals and nearly 500 local pharmacies operating as clinical bases [3] at the beginning of this program. All local pharmacies were supplied with the antiviral drugs after October 30, 2009 [1].

The antiviral treatment guidelines for suspected cases were defined as influenza-like illness (temperature ≥ 37.8°C, with at least one of the following symptoms: rhinorrhea, nasal congestion, sore throat, or cough) and one of following subgroups [7]: 1. high-risk groups such as young children ≤ 59 months, women who were pregnant or 2 weeks postpartum, elderly ≥ 65 years and people with chronic illnesses (pulmonary disease, cardiovascular disease, diabetes, kidney disease, liver disease, malignancy, immune suppression, and others such as cognitive disorders, spinal damage, and neuromuscular disorders), 2. medical personnel previously in contact with a patient with confirmed or suspected infection, 3. admitted patients, and 4. cases diagnosed as necessary based on a doctor’s decision.

**Figure 1:**
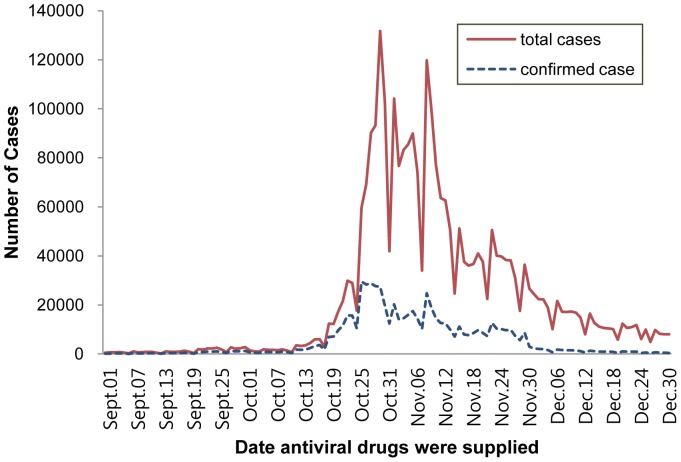
Number of antiviral drug users in the Antiviral Drug Surveillance System (ADSS) from September to December 2009. The frequency dropped at the time of clinic and pharmacy closings on Sundays.

According to the Korean Influenza Surveillance Scheme, influenza-like illnesses exceeded the 2009 baseline (2.6/1000) [8] at the pandemic level during week 34 (August 16–22), sharply increased beginning on week 42 (October 11–17), and peaked during week 45 (November 1–7) at 44.96/1,000 outpatients [9]. Similarly, the number of antiviral prescriptions peaked during weeks 44–46 (October 25–November 15) (Fig. 1).

Information from the ADSS consisted of gender, age, region, date of prescription, and dispensing pattern: outpatient, inpatient, or intensive care unit (ICU). We classified patients who had a lab-confirmed recoding on the national health insurance claim during the study period as a confirmed case. Novel influenza A (H1N1) infection was confirmed in Korea by real-time reverse transcription polymerase chain reaction (RT-PCR) analysis or by conventional RT-PCR at the Research Institute of Public Health and Environment in each province or at a medical center capable of laboratory testing [10].

We assessed economic status according to the type of beneficiary, either covered by National Health Insurance (NHI) or by the Medical Aid program, a Korean public assistance program. In 2008, 96.3% of the total population was covered by the NHI; the remaining individuals (3.7%) were indigent or in lower income brackets and were covered by the Medical Aid program [11].

Various “underlying diseases” were identified from the diagnosed health benefit claim codes for patients from September 1, 2008 to August 31, 2009, 1 year prior to the study period. The underlying conditions were classified into pulmonary disease, cardiovascular disease, diabetes, kidney disease, liver disease, malignancy, immune suppression, and others such as cognitive disorders, spinal damage, and neuromuscular disorders as mentioned in the antiviral treatment guidelines.

We assumed a case to be a death associated with novel influenza A (H1N1) when a patient with a lab-confirmed record during the study period lost beneficiary eligibility due to death as of December 31, 2009. Data of body mass index (BMI) and smoking and drinking habits for adults aged ≥ 20 yr, who were part of the study population, were collected from the 2008 and 2009 Periodic Health Examination Program (PHEP) records. PHEP is a free-of-charge service benefit for NHI beneficiaries who are householders, employees, or dependents of these two groups aged ≥ 40 yr. The National Health Insurance Cooperation (NHIC) suggests that every recipient under the category receive the service at least biannually, and 66% of those recipients received medical examinations in 2009 [12].

Patient confidentiality was maintained through the use of unidentified data forms from the NHIC, where all national health benefits are managed and where ADSS was operated during the pandemic. The initial data source was part of the routinely collected information by NHIC for administrative purposes, and the ADSS dataset was reconstructed without personal identification revealed to monitor demand for the antiviral drugs. The Institutional Review Board (IRB) of the School of Public Health, Seoul National University waived the need for written informed consent from the participants, because no patient identification information was included in the dataset. This decision was based on the “protection of study participants” regulation of the IRB of the School of Public Health, Seoul National University.

### Statistical Analysis

We used descriptive analyses of cases by gender, age, health benefit, region, and the presence or absence of an underlying disease. Means (± standard deviation) and medians of continuous variables and percentages of categorical variables were generated. Multiple logistic regressions were used to identify independent risk factors of disease severity, and the results are expressed as odds ratios (ORs) and 95% confidence intervals (CIs). The disease severities we considered in this study were the proportion of severe outcomes such as general admissions or admissions to the ICU. Complementary analyses were carried on confirmed cases out to examine results consistency. We included variables of the month and day of the week to correct for changes in the sensitivity and specificity of clinical surveillance schemes throughout the epidemic and for accessibility to clinics according to when they were open. All statistical analyses were performed with SAS 9.1 (SAS Institute, Cary, NC, USA) and Microsoft Excel (Redmond, WA, USA).

## Results

### Epidemiological Characteristics

In total, 2,825,821 antiviral drug users were registered in the ADSS from September 1 to December 31, 2009, including 665,231 confirmed cases (Table 1). More than 50% of the patients were reported in and around the Korean capital area. A total of 716,922 patients were from Kyonggi Province, 547,441 were from Seoul, and 156,035 were from Incheon. Females accounted for 50.02% of all patients and 52.66% of confirmed cases. The mean age was 19.9 yr (±17.3 yr) and the median age was 14 yr (range, 0–102 yr). Substantially more cases were recorded in the younger group than those in the older group. Children aged 0–9 yr accounted for 33.94% of all cases, whereas only 3.89% of the patients were ≥ 60 yr. The school-age group of 10–19 yr had the highest number of confirmed cases. A total of 759,165 (26.9%) patients had one or more underlying medical comorbidities, and 59.24% of these had lung disease including asthma, and 65.12% of the patients with lung disease were ≤ 9 yr. Liver disease was equally distributed over all age groups. The incidences of diabetes and malignancy increased with age. The distribution of the types of disease for confirmed cases was similar to the distribution for all cases.

**Table 1:** Characteristics of patients registered in the Antiviral Drug Surveillance System in Korea (September-December 2009).

Characteristics	Total case (%) N = 2825821	Confirmed case (%) N = 665231
**Female sex**	1413423 (50.02)	350458 (52.66)
**Age, yr (Mean, Median)**	(19.9 ± 17.3, 14)	(16 ± 13, 13)
0–4	415854 (14.72)	77277 (11.62)
5–9	543088 (19.22)	160049 (24.06)
10–19	870002 (30.79)	263048 (39.54)
20–29	305766 (10.82)	68740 (10.33)
30–39	281657 (9.97)	44647 (6.71)
40–49	185747 (6.57)	26368 (3.96)
50–59	113698( 4.02)	15608 (2.35)
60+	110009 (3.89)	9494 (1.43)
**Health benefit, Insurance**	2737755 (96.88)	645191 (96.99)
**Region, Province** *	1560224 (55.22)	345593 (51.96)
Seoul†	547441 (19.37)	131895 (19.83)
Pusan†	181792 (6.43)	52205 (7.85)
Taegu†	129533 (4.58)	27636 (4.15)
Inchon†	156035 (5.52)	37862 (5.69)
Kwangju†	95661 (3.39)	22861 (3.44)
Taejon†	87245 (3.09)	24375 93.66)
Ulsan†	67454 (2.39)	22731 (3.42)
Kyonggi*	716922 (25.37)	157670 (23.70)
Gangwon*	84992 (3.01)	30476 (4.58)
Chungbuk*	97465 (3.45)	21886 (3.29)
Chungnam*	114198 (4.04)	24065 (3.62)
Chonbuk*	105107 (3.72)	22107 (3.32)
Chonnam*	90440 (3.20)	17759 (2.67)
Kongbuk*	129076 (4.57)	25711 (3.86)
Kongnam*	201173 (7.12)	42541 (6.39)
Jeju*	20851 (0.74)	3378 (0.51)
**≥ 1 underlying disease**	n = 759165 (26.9)	n = 160377 (24.1)
Lung disease	529398 (59.24)	120248 (66.66)
Cardiovascular disease	63610 (7.12)	9240 (5.12)
Diabetes mellitus	61529 (6.89)	7396 (4.10)
Kidney disease	23420 (2.62)	4620 (2.56)
Liver disease	103358 (11.57)	18348 (10.17)
Malignancy	25885 (2.9)	3576 (1.98)
Immune suppression	50057 (5.6)	9978 (5.53)
others	36322 (4.06)	6978 (3.87)

**NOTE**. ^†^Seven large cities in Korea.

* Nine provinces in Korea.

**Figure 2:**
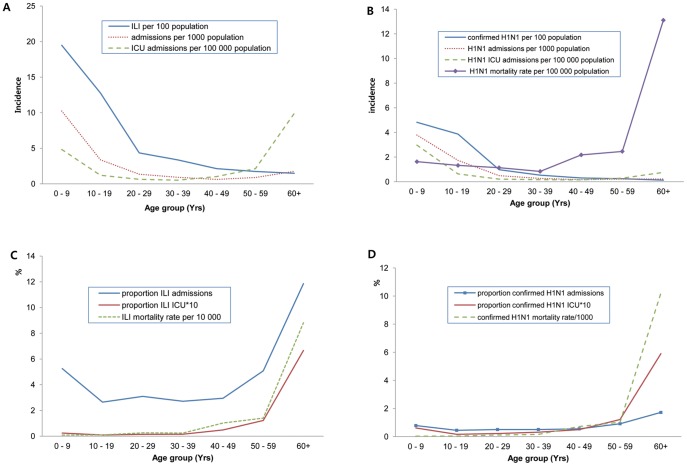
Epidemic curves of confirmed or suspected influenza A (H1N1) (A) and confirmed only (B). Proportion of severe outcomes among confirmed or suspected influenza A (H1N1) cases by age group (C) and confirmed cases only (D).

Most of the cases in Korea from September to December 2009 were community infections [13]. The estimated incidence of novel influenza A (H1N1) during the pandemic was 5.68/100 for all cases, and 1.34/100 were confirmed cases. Infection occurred mostly in the younger age groups (Fig. 2A), and the 5–9 yr age group being the most affected (20.42/ 100), whereas the ICU admission rates were similar for patients in the 0–9 yr group and in the group ≥ 60 yr (Table 2). We counted a case as a death when the patient was confirmed as having the infection but was dead at the end of the study period. The mortality due to influenza A (H1N1) using this criterion was 0.33/100,000, with the highest mortality of 1.31/100,000 for patients ≥ 60 yr. The estimated incidence, admission rate, and ICU rate in the confirmed group were highest in the younger groups and decreased gradually in the older groups (Fig. 2B).

**Table 2:** Estimating the incidence of novel influenza A (H1N1) by age group and region during September to December 2009 in Korea.

Age(yrs)	population	Total cases†	Admission/1000	ICU /100000	confirmed /100	confirmed admission /1000	confirmedICU /100000	H1N1 Mortality /100000
0–4	2263425	18.37	13.08	4.64	3.41	3.27	1.86	0.22
5–9	2659544	20.42	7.82	5	6.02	4.18	3.91	0.11
10–19	6811650	12.77	3.38	1.2	3.86	1.73	0.63	0.13
20–29	7028262	4.35	1.35	0.64	0.98	0.49	0.18	0.11
30–39	8371533	3.36	0.91	0.51	0.53	0.27	0.16	0.08
40–49	8729495	2.13	0.63	1.03	0.3	0.17	0.11	0.22
50–59	6508077	1.75	0.89	2.14	0.24	0.22	0.29	0.25
60+	7401159	1.49	1.76	9.9	0.13	0.21	0.62	1.31
**total**	49773145	5.68	2.31	2.75	1.34	0.81	0.58	0.33
**Region**								
**City**	22984378	5.5	2.23	2.28	1.39	0.88	0.53	0.28
Seoul	10208302	5.36	2.49	3.07	1.29	0.96	0.8	0.18
Pusan	3543030	5.13	2.65	1.41	1.47	1.14	0.25	0.51
Taegu	2489781	5.2	1.21	0.72	1.11	0.42	0.16	0.32
Inchon	2710579	5.76	1.75	2.36	1.4	0.67	0.3	0.26
Kwangju	1433640	6.67	1.07	1.46	1.59	0.36	0.28	0.07
Taejon	1484180	5.88	3.4	2.96	1.64	1.15	0.34	0.4
Ulsan	1114866	6.05	1.87	1.26	2.04	1.06	0.36	0.54
**Province**	23788767	5.82	2.37	3.15	1.29	0.76	0.69	0.37
Kyonggi	11460610	6.26	2.52	3.16	1.38	0.82	0.56	0.28
Gangwon	1512870	5.62	2.05	4.89	2.01	0.72	1.12	0.93
Chungbuk	1527478	6.38	2.05	3.54	1.43	0.63	0.39	0.46
Chungnam	2037582	5.6	1.71	2.7	1.18	0.48	0.29	0.49
Chonbuk	1854508	5.67	1.35	1.35	1.19	0.57	0.16	0.16
Chonnam	1913004	4.73	1.96	1.73	0.93	0.6	0.21	0.63
Kongbuk	2669876	4.83	2.3	3.48	0.96	0.67	0.3	0.37
Kongnam	3250176	6.19	3.46	4.25	1.31	1.05	2.03	0.37
Jeju	562663	3.71	2.35	1.6	0.6	0.85	0	0

**NOTE**. Incidence  =  no. of each cases ÷ population of each age group.

† All patients registered in the Antiviral Drug Surveillance System (ADSS) were confirmed or suspected to have the infection.

The number of patients exposed to novel influenza A (H1N1) was highest in and around the capital area, but the incidence per 100 people was high in Gwangju (6.67) and Chungbuk (6.38). The highest incidence rate of severe outcomes was in Gangwon (4.89 ICU admissions/100,000), where most of the districts are rural areas. After classifying the region by city and province, the incidence of influenza A (H1N1) was higher in provinces where the proportions of 0–19 yr patients (24.30%) and those ≥ 60 yr (15.99% ) were greater than those in the city (22.73% and 13.57% respectively).

### Factors Associated with Severe Outcomes

Of the total antiviral drug users in the ADSS, 2,709,611 (95.9%) were outpatients, 114,840 (4.06%) were inpatients, and 1,370 (0.05%) were admitted to ICUs. Females comprised 49.86% of the outpatients, 53.57% of the inpatients, and 61.17% of the ICU patients. ORs increased with disease severity in the multivariate analyses (Table 3). The average age of the outpatients was 19.8 yr (±16.9 yr) and the median was 14 yr (range, 0–102 yr). The mean and median ages increased to 51.6 (±28.5 yr) and 62 yr (range, 0–96 yr), respectively, for those in the ICU. Compared to those aged 30–39 yr, those ≥ 60 yr were significantly more likely to have a severe outcome (ICU; OR, 30.988; 95% CI, 22.594–42.501). The proportion of NHI beneficiaries was 96.68% for outpatients, but this value decreased to 94.77% and 89.12% for general and ICU admissions, respectively. NHI beneficiaries were less likely to experience severe illness than patients in the Medical Aid program (ICU; OR, 0.460; 95% CI, 0.387–0.548). Underlying disease was associated with an increased risk of severe outcome. The OR was 1.280 (95% CI, 1.263–1.297) for inpatients and 2.065 (95% CI, 1.829–2.332) for those admitted to the ICU.

**Table 3:** Multivariate factors associated with a severe outcome in relation to a nonsevere outcome among all antiviral drug users.

Characteristics	Outpatients No.(%) n = 2709611	Inpatients No.(%) n = 114840	OR (95% CI)	ICU No.(%) n = 1370	OR (95% CI)
**Female sex**	1351062 (49.86)	61523 (53.57)	1.165 (1.151–1.179)	838(61.17)	1.996 (1.786–2.231)
**Age (yrs)**(Mean, Median)	(19.8±16.9, 14)	(22±23.4, 13)		(51.6±28.5, 62)	
0–4	386140(14.25)	29,609(25.78)	2.519 (2.453–2.587)	105(7.66)	1.283 (0.896–1.838)
5–9	522150(19.27)	20805(18.12)	1.359 (1.322–1.336)	133(9.71)	1.443 (1.020–2.040)
10–19	846901(31.26)	23019(20.04)	0.931 (0.907–0.957)	82(5.99)	0.641 (0.442–0.930)
20–29	296259(10.93)	9462(8.24)	1.152 (1.117–1.188)	45(3.28)	0.985 (0.649–1.497)
30–39	273967(10.11)	7647(6.66)	reference	43(3.14)	reference
40–49	180175(6.65)	5482(4.77)	1.030 (0.994–1.067)	90(6.57)	3.016 (2.096–4.339)
50–59	107784(3.98)	5775(5.03)	1.648 (1.590–1.708)	139(10.15)	6.580 (4.660–9.290)
60+	96235(3.55)	13041(11.36)	3.575 (3.463–3.692)	733(53.50)	30.988 (22.594–42.501)
**Health benefit, Insurance**	2627703(96.68)	108831(94.77)	0.585 (0.569–0.602)	1221(89.12)	0.46 (0.387–0.548)
**Region, Province**	1495874(55.21)	63507(55.33)	0.977 (0.965–0.989)	843(61.67)	1.311 (1.175–1.463)
**≥1 underlying disease** †	n = 713383(26.33)	n = 44887(39.09)	1.28 (1.263–1.297)	n = 895(65.33)	2.065 (1.829–2.332)
Lung disease	498284(59.87)	30670(51.36)	1.169 (1.152–1.186)	444(28.59)	1.493 (1.326–1.682)
Cardiovascular disease	57398(6.90)	5929(9.93)	1.286 (1.247–1.327)	283(18.22)	1.531 (1.325–1.768)
Diabetes mellitus	55435(6.66)	5804(9.72)	1.256 (1.216–1.297)	290(18.67)	1.401 (1.214–1.617)
Kidney disease	20996(2.52)	2342(3.92)	1.801 (1.720–1.885)	82(5.28)	2.049 (1.619–2.584)
Liver disease	97918(11.76)	5322(8.91)	1.037 (1.006–1.068)	118(7.60)	0.740 (0.609–0.899)
Malignancy	22232(2.67)	3489(5.84)	2.298 (2.208–2.391)	164(10.56)	2.526 (2.123–3.006)
Immune suppression	46515(5.59)	3438(5.76)	1.344 (1.295–1.395)	104(6.70)	1.909 (1.549–2.352)
others	33533(4.03)	2721(4.56)	1.436 (1.378–1.496)	68(4.38)	1.502 (1.171–1.927)

**NOTE**. Odds ratios (ORs) were adjusted with eight categories of underlying disease.

† Results for multivariate logistic regression without considering the various underlying diseases.

Confirmation rates differed by age group in a subset of lab-confirmed cases. The majority (75.22%) of confirmed patients was < 20 yr, and the confirmation rates were high in school-aged individuals, with the highest at 30.24/100 cases for those aged 10–19 yr. Only 3.89% of confirmed cases were elderly (≥60 yr), and their confirmation rate was the lowest at 8.63/100 cases. Analyses restricted to lab-confirmed cases showed similar results, with the ORs of those ≥ 60 yr higher than those of the younger groups, but the magnitude of the ORs was reduced compared with ORs in all cases (Table 4).

**Table 4:** Multivariate factors associated with a severe outcome in relation to a nonsevere outcome in lab-confirmed cases.

Characteristics	Outpatients No.(%)n = 624330	Inpatients No.(%)n = 40,539	OR	95% CI	ICU No.(%) n = 290	OR	95% CI
**Female sex**	327892 (52.51)	22225 (54.82)	1.116	(1.093–1.139)	196 (64.05)	1.670	(1.257–2.220)
**Age (yrs)**	(16±13, 13)	(17±17, 11)			(24±26, 9)		
0–4	69833 (11.18)	7402 (18.26)	1.781	(1.694–1.873)	42 (13.73)	1.800	(0.820–3.952)
5–9	148813 (23.83)	11132 (27.46)	1.318	(1.257–1.382)	104 (33.99	2.348	(1.120–4.921)
10–19	251228 (40.24)	11777 (29.05)	0.841	(0.802–0.881)	43 (14.05)	0.743	(0.339–1.627)
20–29	65265 (10.45)	3460 (8.53)	1.002	(0.949–1.059)	15 (4.90)	0.694	(0.260–1.851)
30–39	42379 (6.79)	2254 (5.56)	reference		14 (4.58)	reference	
40–49	24901 (3.99)	1454 (3.59)	1.032	(0.964–1.105)	13 (4.25)	1.110	(0.384–3.210)
50–59	14162 (2.27)	1427 (3.52)	1.626	(1.515–1.746)	19 (6.21)	3.308	(1.346–8.133)
60+	7805 (1.25)	1633 (4.03)	2.929	(2.719–3.156)	56 (18.30)	10.129	(4.380–23.423)
**Health benefit, Insurance**	606123 (97.08)	38782 (95.67)	0.659	(0.626–0.694)	286 (93.46)	0.687	(0.370–1.278)
**Region, Province**	325043 (52.06)	20365 (50.24)	0.921	(0.902–0.940)	185 (60.46)	1.520	(1.148–2.011)
**≥1 underlying disease** †	146481 (23.46)	13,737 (33.89)	1.328	(1.298–1.359)	159 (51.96)	2.029	(1.516–2.717)
Lung disease	109708 (67.02)	10422 (63.29)	1.287	(1.255–1.319)	118 (49.79)	2.069	(1.541–2.779)
Cardio. disease	8080 (4.94)	1,132 (6.87)	1.312	(1.224–1.407)	28 (11.81)	1.887	(1.083–3.288)
Diabetes	6413 (3.92)	950 (5.77)	1.286	(1.189–1.391)	33 (13.92)	2.273	(1.286–4.017)
Kidney disease	4123 (2.51)	491 (2.98)	1.461	(1.326–1.611)	6 (2.53)	1.282	(0.466–3.529)
Liver disease	17012 (10.39)	1319 (8.01)	1.043	(0.982–1.107)	17 (7.17)	1.057	(0.557–2.005)
Malignancy	3047 (1.86)	518 (3.15)	1.764	(1.596–1.950)	11 (4.64)	2.441	(1.177–5.061)
Immune supp.	9062 (5.54)	901 (5.47)	1.279	(1.191–1.375)	15 (6.33)	2.137	(1.130–4.044)
others	6234 (3.81)	735 (4.46)	1.463	(1.351–1.584)	9 (3.78)	1.558	(0.682–3.558)

**NOTE**. Odd ratios (ORs) were adjusted with eight categories of underlying disease.

† Results for multivariate logistic regression without considering the various underlying diseases.

### Likelihood of Death

Although the incidence and admission rate for influenza A (H1N1) were higher in younger individuals, the proportions of inpatients and those admitted to the ICU among antiviral drug users were higher in the elderly (≥ 60 yr) (Fig. 2C, 2D) and the mortality rate for those ≥ 60 yr was noticeably higher than that in other groups. The death rate significantly differed by the time the prescription was filled with 0.01/100 for outpatients and 0.23 and 5.23/100 for admission and ICU, respectively. Because the stage that the drugs were used influenced mortality, we adjusted the ORs for death including the variable for the time of filling the prescription. Compared to those aged 30–39 yr, those ≥ 60 yr were significantly more likely to die (OR, 20.747; 95% CI, 9.2874–46.348). Meanwhile, the risks of the younger group were much lower (0–4 yr; OR 0.317; 95% CI, 0.099–1.010; 5–9 yr, OR. 0.106; 95% CI, 0.027–0.411).

### Behavioral Variables

Registered patients ≥ 20 yr old in the biannual PHEP data numbered 397,390 among the total study population. Of those, 14,876 patients were hospitalized, and 258 were admitted to the ICU (Table 5). Approximately 71% were aged 20–49 yr but 84.50% of ICU patients were ≥ 50 yr. The proportion of individuals with underlying diseases in the BMI subgroup was higher than that in the total group. Underweight (BMI < 18.5****kg/m^2^) patients had a higher risk of severe illness. Although the number of high BMI patients was greater than the number of low BMI patients for all outcomes, the proportion of obese patients (BMI > 25.0****kg/m^2^) decreased, whereas the proportion of low BMI patients increased as the infection became more severe (Table 5). The adjusted ORs for underweight patients were significantly different from the ORs of patients whose weight was normal. For all patients ≥ 20 yr, the ORs were 1.436 (95% CI, 1.334–1.546) for inpatients and 2.953 (95% CI, 1.830–4.767) for those admitted to the ICU (Table 6). Although the cases in variable categories with behavior variables among confirmed cases were not sufficient for a precise analysis, the trends in the ORs remained the same in confirmed cases. The ORs for low BMI patients were 1.189 (95% CI, 1.024–1.379) for inpatients and 2.387 (95% CI, 0.827–6.894) for those admitted to the ICU or who died. All ORs were adjusted with other variables such as gender, age, region, and underlying condition.

**Table 5:** Baseline characteristics of the body mass index (BMI) subset.

	Total No.(%) (n = 397,390)		Confirmed No.(%) (n = 66,912)			
Characteristics	Outpatients (n = 382,256)	Inpatients (n = 14,876)	ICU (n = 258)	Outpatients (n = 63,058)	Inpatients(n = 3,830)	ICU (n = 24)
**Sex, female**	187,023 (48.93)	7,449 (50.07)	180 (69.77)	32,442 (51.45)	2123 (55.43)	10 (41.67)
**Age (yrs)**						
20–29	83,978 (21.97)	2,521 (16.95)	8 (3.10)	19,454 (30.85)	911 (23.79)	3 (12.50)
30–39	109,830 (28.73)	3,129 (21.03)	11 (4.26)	19,263 (30.55)	923 (24.10)	4 (16.67)
40–49	79,851 (20.89)	2,138 (14.37)	21 (8.14)	11,722 (18.59)	632 (16.50)	1 (4.17)
50–59	63,447 (16.60)	2,739 (18.41)	38( 14.73)	8,784 (13.93)	738 (19.27)	5 (20.83)
60+	45,150 (11.81)	4,349 (29.24)	180 (69.77)	3,835 (6.08)	626 (16.34)	11 (45.83)
**BMI(kg/m^2^)**	23.48±3.43			23.29±3.49		
Underweight (BMI<18.5)	20,613 (5.39)	1,029 (6.92)	25 (9.69)	3,889 (6.17)	251 (6.55)	1 (4.17)
Normal (18.5≤BMI<25.0)	245,678 (64.27)	9,448 (63.51)	160 (62.02)	41,276 (65.46)	2,430 (63.45)	15 (62.50)
Obese (25.0≤BMI)	115,965 (30.34)	4,399 (29.57)	73 (28.29)	17,893 (28.38)	1,149 (30.00)	8 (33.33)
**Smoking†**	90,171 (25.26)	3,176 (23.22)	57 (25.56)	15,461 (26.03)	805 (22.52)	7 (33.33)
**Drinking** ‡	63,152 (18.71)	2,121 (16.27)	31 (14.49)	10,762 (19.19)	547 (16.02)	2 (10.00)
**Region, province**	203,347 (53.20)	8,268 (55.59)	169 (65.50)	32,091 (50.89)	1,857 (48.49)	15 (62.50)
**underlying disease**	113,344 (29.65)	6,633 (44.59)	189 (73.26)	15,726 (24.94)	1,403 (36.63)	13 (54.17)
Lung disease	42081	2806	88	5812	609	9
Cardiovascular disease	21248	1594	69	2548	297	5
Diabetes mellitus	23755	1781	63	2653	327	6
Kidney disease	4744	445	10	679	6	1
Liver disease	34396	1643	34	5051	368	2
Malignancy	8011	973	56	1058	142	2
Immune suppression	10782	682	9	1606	170	2
others	7850	472	10	1048	98	0

**NOTE.** ^†^current smoker.

‡ drink more than once or twice per week.

**Table 6:** Multivariate behavioral predictors associated with a severe outcome in relation to a nonsevere outcome among all cases.

	Total (N = 397,390)	
Characteristics	Inpatients, OR(95% CI)	ICU, OR(95% CI)
Female sex	1.117 (1.070–1.165)	2.601 (1.883–3.591)
Age (yrs)		
20–29	1.046 (0.987–1.108)	0.922 (0.327–2.600)
30–39	reference	reference
40–49	0.952 (0.896–1.012)	2.793 (1.219–6.400)
50–59	1.435 (1.351–1.523)	6.266 (2.927–13.417)
60+	2.829 (2.670–2.998)	31.021 (15.382–62.562)
**BMI** (kg/m^2^)		
**Underweight (BMI<18.5)**	**1.436 (1.334–1.546)**	**2.953 (1.830–4.767)**
**Normal (18.5≤BMI<25.0)**	**reference**	**reference**
**Obese (25.0≤BMI)**	**0.903 (0.867–0.941)**	**0.840 (0.614–1.150)**
Smoking†	1.052 (1.000–1.107)	1.305 (0.907–1.879)
Drinking‡	0.959 (0.911–1.010)	0.789 (0.515–1.209)
region, province	1.041 (1.004–1.080)	1.503 (1.120–2.017)
≥1 underlying disease	1.428 (1.372–1.486)	2.378 (1.712–3.302)
Lung disease	1.367 (1.302–1.436)	1.734 (1.299–2.383)
Cardiovascular disease	1.192 (1.118–1.272)	1.937 (1.380–2.350)
Diabetes Mellitus	1.218 (1.145–1.296)	1.249 (0.885–1.764)
Kidney disease	1.739 (1.552–1.949)	1.146 (0.531–2.473)
Liver disease	1.029 (0.969–1.094)	0.785 (0.505–1.221)
Malignancy	2.076 (1.915–2.251)	4.274 (2.9945–6.100)
Immune supp.	1.280 (1.169–1.400)	1.575 (0.914–2.714)
others	1.139 (1.024–1.266)	0.905 (0.422–1.942)

**NOTE.** ^†^current smokers.

‡ drink more than once or twice per week.

## Discussion

During the study period from September–December 2009, 5.69% of the Korean population was prescribed antiviral drugs and 2.3/1,000 people were admitted as confirmed or suspected cases of infection. The proportion of females was higher among severe infection cases. A dominant prevalence of female cases was also reported in Canada [14]. However, a gender-specific infection could not be concluded clearly, because other variables associated with females, such as pregnancy, [15], [16] were not included in the present analyses.

Kim *et al*. (2010) [17] studied the trend of the spread of this novel influenza strain by comparing three monitoring tools used in Korea during the pandemic. The patterns of spread from the three methods were generally similar but details, such as peak time, were different. We found that illness severity was greater among patients who were ≥ 60 yr, who were in a low-income group, and who had comorbidities. This finding persisted in the results for analysis of the confirmed group only.

Most previous studies have reported the characteristics of novel influenza A (H1N1) lab-confirmed cases. However, as novel influenza A (H1N1) became a pandemic, routine testing for the infection was not recommended, and prompt treatment was given instead to mitigate damage from the infection. Therefore, an analysis of only confirmed cases would certainly lead to selection bias in the results. Because the entire population that was given antiviral drugs, including those that were treated during the peak period of the pandemic, were considered in this study, we were able to conclude that there was no difference between the results for all cases and the results for only confirmed cases. This increases the confidence of our findings.

The age-specific immunity of novel influenza A (H1N1) reported in a previous study by Miller *et al*. (2010) [18] suggested that a great portion of older people had pre-existing immunity to the novel influenza A (H1N1) virus as a result of pre-exposure to antigenically related influenza A earlier in their lifetime. Moreover, the significantly higher incidence of infection in those ≤ 19 yr occurred presumably because these patients were exposed to an increased potential for transmission within their schools, and confirmation tests were performed vigorously [19]. In our study, the characteristics of novel influenza A (H1N1) appeared to be age-specific in terms of mortality. Younger aged individuals were susceptible to the influenza A (H1N1) infection and had a higher incidence of severe outcomes than those in other age groups. But, the persons in the elderly group (≥60 yr) were much more likely to suffer a severe case or even more fatal cases once they became infected. Schools in Korea were not closed nationwide during the novel influenza A (H1N1) outbreak, and the decision was left to the discretion of each school.

The incidence and admission rate in the younger age group of outpatients and inpatients were higher than those in the older group. ICU incidence was high in the groups < 10 and ≥ 60 yr. Considering that the prevalence was significantly lower in the older group, the risk of severity was assumed to be higher in those aged ≥ 60 yr. This result is consistent with our supplementary analysis of confirmed cases. The incidences in children, who were confirmed patients classified as outpatients, inpatients, and those admitted to the ICU, were higher than those in the older group, but an exception was found for deaths assumed to be caused by novel influenza A (H1N1), which were higher in the older group than those in children. The mortality rate showed a J-shaped curve with the greatest risk in those aged ≥ 60 yr (1.31/100,000). This finding can be interpreted as most young children recovered from the severe illness, whereas severe illness more often led to death in the older group.

A risk factor associated with obesity was not found in Korea, which differed from results reported in Mexico [20] and the United States [21]. However, being underweight was one of the risk factors for severity in our study. The reason for the reverse association between BMI and severe outcome is unclear. We used the general cut-off point of BMI as recommended by the WHO. However, Asian populations have different associations between BMI, the percentage of body fat, and health risks compared to Western populations [22]. Blumentals et al. [23] identified the highest influenza pneumonia rates in underweight individuals in their retrospective cohort study of UK patients and suggested an association between low BMI, malnutrition, and immune function. This was also most likely influenced by the observation that a substantial proportion of patients with a severe underlying disease such as cardiovascular disease or chronic pulmonary disease have a lean body mass in general [24]. One of the limitations of this study was that information regarding the severity of the individual underlying diseases did not exist.

We showed various outcomes in the incidence and mortality among regions. Regional variations in illness magnitude may have been caused by the density and composition of the population. Approximately 49% of the Korean population lives in the capital area (Seoul) and around this area, including the city of Incheon and Kyonggi province from among the 16 cities and provinces in Korea. One social issue in Korea is that the average age of the population in rural areas is increasing; thus, it is assumed that age-specific immunity and mortality were the cause of the observed variations in incidence in the regions, together with differences in the transmission potential according to population density. After classifying the region into two groups such as city and province, the incidence of influenza A (H1N1) and the risk of severe outcomes were higher in provinces. The proportion of working people aged in their 20 s to 50 s among residents, the lower risk groups for influenza A (H1N1), was greater in the city.

We found that individual economic status influenced infection severity. Although only two groups were used in this study, consistent results were found throughout the analysis. Patients in the Medical Aid program showed greater disease severity. Accessibility to medical treatment and hygiene could differ according to individual economic conditions. This may have caused a delay in seeking medical care after symptom onset. The length of time from symptom onset to treatment is associated with illness severity [25].

Underlying medical conditions are a risk factor for severe influenza [26]. Echevarria-Zuno *et al*. (2009) [20] reported that the presence of an underlying disease increased the risk of dying to by 6.1 fold in Mexico. We found that the OR for death cases with underlying disease was 2.218 (95% CI, 1.504–3.271) adjusted by all other variables including the phase of prescription refill.

Our study is an aggregated case report including almost all cases of confirmed and suspected infection during and around the pandemic peak. Individual case reports at an early stage of a pandemic are important to make appropriate policy decisions. However, while such reports at the early stage of a pandemic can explain groups susceptible to transmission; they cannot help identify risk groups in the total population. Moreover, these data cannot predict the degree of severity, because the aim of hospitalization at this time is isolation in general [27]. Including the probable cases of novel influenza A (H1N1) in this study would not likely result in overestimating the incidence rate considering that novel influenza A (H1N1) is a relatively mild infection [28] or even an asymptomatic infection for which a majority of cases were not captured [29].

Our study had several limitations. Given that we used data from the ADSS, there was an absence of detailed clinical symptom information. Data related to the type of medication may be limited in its ability to reflect the true conditions of the infection. Another limitation was that prescription information was entered by staff at hundreds of clinics across the county, which may have reduced reliability of the data, but antiviral drugs distributed from national stores were counted and rechecked by the district health center to verify their use and the number of remaining drugs. We were also unable to gather information on underlying disease severity, which precluded a conclusion as to which type of underlying disease most influenced outcomes.
